# High Prevalence of Multidrug-Resistant Bacterial Colonization Among Patients and Healthcare Workers in a Rural Ethiopian Hospital

**DOI:** 10.3390/antibiotics14070717

**Published:** 2025-07-17

**Authors:** Elena Hidalgo, Teresa Alvaredo-Carrillo, Josefina-Marina Gil-Belda, Clara Portela-Pino, Clara Bares-Moreno, Sara Jareño-Moreno, Paula de la Fuente, Lucía Platero, Ramón Pérez-Tanoira

**Affiliations:** 1 Servicio de Microbiología Clínica, Hospital Universitario de Getafe, 28905 Getafe, Spain; 2 Pediatrics Department, King Fahd Lamu County Referral Hospital, Lamu 80500, Kenya; t.alvaredo@outlook.es; 3 Servicio de Urgencias Generales, Hospital Universitario Infanta Elena, 28342 Madrid, Spain; josefina.gil@quironsalud.es; 4 Servicio de Medicina Interna, Complexo Hospitalario Universitario de Ourense, 32005 Ourense, Spain; clara.portela.pino@sergas.es; 5 Medicina Interna, Hospital San Juan Despí Moisés Broggi, 08970 Barcelona, Spain; bares@clinic.cat; 6 Tropical Medicine and International Health, Autonomous University of Madrid, 28049 Madrid, Spain; sarajmoreno88@gmail.com; 7 Servicio de Urgencias Generales, Hospital San Agustín, 33401 Asturias, Spain; pauladelafuenteg@gmail.com; 8 Servicio de Medicina Interna, Hospital Universitario de la Paz, 28046 Madrid, Spain; lucia.platero@salud.madrid.org; 9 Servicio de Microbiología Clínica, Hospital Universitario Príncipe de Asturias, 28805 Alcalá de Henares, Spain; 10 Department of Biomedicine and Biotechnology, Faculty of Medicine, University of Alcalá, 28871 Alcalá de Henares, Spain

**Keywords:** nasal colonization, gastrointestinal colonization, multidrug resistant bacteria, methicillin-resistant *Staphylococcus aureus*, vancomycin-resistant *Enterococcus* spp., carbapenemases, Ethiopia

## Abstract

Background/Objectives: Multidrug-resistant (MDR) bacterial colonization poses a significant risk for subsequent infections, especially within hospital environments. Healthcare workers can inadvertently transmit these MDR bacteria to vulnerable patients, exacerbating the problem. This study aimed to determine the colonization rates of MDR bacteria among patients and healthcare workers in a rural Ethiopian hospital with limited resources. Methods: Between 26 May and 6 June 2024, nasal, rectal, vagino-rectal exudate, and stool samples were collected from patients (*n* = 78) and healthcare workers (*n* = 11) at Gambo General Hospital (Oromia Region, Ethiopia). Samples were cultured on chromogenic media selective for methicillin-resistant *Staphylococcus aureus* (MRSA), vancomycin-resistant *Enterococcus* spp. (VRE), and carbapenemase-producing *Enterobacteriaceae* (CPE). Bacterial identification was performed using MALDI-TOF mass spectrometry (Bruker), antimicrobial susceptibility testing using the MicroScan WalkAway system (Beckman Coulter), and genotypic characterization with the MDR Direct Flow Chip kit (Vitro). Results: MRSA nasal colonization was detected in 43.3% of patients (13/30; 95% CI: 27.4–60.8%) and 27.3% of healthcare workers (3/11; 95% CI: 6.0–61.0%) (*p* = 0.73). Rectal (or stool) colonization by MDR bacteria was significantly higher in pediatric patients (85.0%, 17/20; 95% CI: 62.1–96.8%) than in adults (14.3%, 4/28; 95% CI: 5.7–31.5%) (*p* < 0.001). Notably, a high proportion of pediatric patients harbored *Escherichia coli* strains co-producing NDM carbapenemase and CTX-M ESBL, and VRE strains were also predominantly isolated in this group. Conclusions: This study reveals a concerningly high prevalence of MRSA and MDR *Enterobacteriaceae*, especially among children at Gambo Hospital. The VRE prevalence was also substantially elevated compared to other studies. These findings underscore the urgent need for strengthened infection control measures and antimicrobial stewardship programs within the hospital setting.

## 1. Introduction

Antimicrobial resistance (AMR) is a critical global health challenge, with a continuous increase in the prevalence of multidrug-resistant (MDR) microorganisms worldwide [1]. Hospitals serve as hotspots for AMR transmission due to factors such as increased antibiotic use, susceptible patient populations, and challenges in implementing effective infection control measures. The World Health Organization (WHO) has identified priority pathogens for the development of new antibiotics, including Enterobacterales third-generation cephalosporin- and carbapenem-resistant, vancomycin-resistant *Enterococcus faecium*, and methicillin-resistant *Staphylococcus aureus* [2,3]. These pathogens are frequently implicated in both colonization and infection, leading to significant morbidity and mortality, and were included in this study.

Enterobacterales, a component of the intestinal microbiota, are common causes of nosocomial and community-acquired infections. Resistance to beta-lactam antibiotics in Enterobacterales is driven by mechanisms such as efflux pumps, reduced membrane permeability, and the production of beta-lactamases—enzymes that hydrolyze the beta-lactam ring, including extended-spectrum beta-lactamases (ESBLs) and carbapenemases [4]. The genes encoding these enzymes are often located on mobile genetic elements, facilitating their horizontal transfer and co-expression with resistance genes for other antibiotic classes [5,6].

Enterococci are also common colonizers of the intestinal tract, with *E. faecium* and *E. faecalis* being the most common causes of human infections. Glycopeptide resistance in enterococci, mediated by genes *vanA* and *vanB*, has become increasingly prevalent. The VanA phenotype is characterized by the expression of high levels of resistance to vancomycin and teicoplanin, whereas the VanB phenotype shows moderate resistance to vancomycin and susceptibility to teicoplanin [7,8].

*Staphylococcus aureus* colonizes the nasal mucosa of 25–50% of the general population and can act as an opportunistic pathogen causing serious infections [9]. Resistance to beta-lactam antibiotics in *S. aureus* is typically mediated by the *mecA* gene, which encodes a modified penicillin-binding protein [9].

The hospital environment facilitates the transmission of MDR microorganisms due to factors such as susceptible patients, antibiotic use, and a higher prevalence of colonized or infected individuals [10]. Asymptomatic carriers of MDR bacteria can serve as reservoirs for transmission and are at increased risk of subsequent infection [11,12,13,14], highlighting the importance of understanding the population of MDR bacteria in our healthcare settings.

Levels of multidrug resistance have been reported to be higher in low- and middle-income countries (LMICs) compared to high-income countries [15]. Ethiopia faces significant challenges related to AMR, including limited resources and surveillance systems [16]. This is reflected in the high prevalence of MDR bacteria in Ethiopian hospitals [6].

Gambo General Hospital, located in the Oromia region of Ethiopia, is a rural hospital with limited resources, where empiric antibiotic treatment is widely used due to the lack of microbiological infrastructure for bacterial identification and antimicrobial susceptibility testing. In this context, routine surveillance of MDR microorganism’s colonization is not performed, and there is limited awareness of the hospital’s endemic burden of antimicrobial resistance. This study aimed to investigate the colonization rates of MRSA, carbapenemase-producing Enterobacterales, and vancomycin-resistant *Enterococcus* spp. among patients and healthcare workers at Gambo General Hospital. By generating baseline data in a setting where microbiological surveillance is currently absent, this study helps to fill a critical knowledge gap, highlighting that antibiotic resistance is indeed a problem that affects all parts of the world, and may serve as a foundation for future infection control strategies and antimicrobial stewardship interventions in similar resource-limited environments.

## 2. Results

### 2.1. Patient and Sample Characteristics

The study included 41 participants with nasal swab samples (30 patients and 11 healthcare workers) and 48 participants with rectal or stool samples (20 pediatric patients and 28 adults). Summary statistics for age are presented in Table 1.

### 2.2. Nasal Colonization Study

Nasal swabs were collected from 41 participants (30 patients and 11 healthcare workers). MRSA was isolated from 16 samples. Antibiotic susceptibility testing and genotypic analysis were performed on eight MRSA isolates. All isolates were confirmed to be resistant to oxacillin and cefoxitin through phenotypical antibiotic susceptibility testing. Seven isolates exhibited resistance to erythromycin with inducible clindamycin resistance, five were resistant to ciprofloxacin and levofloxacin, and four were resistant to gentamicin and tobramycin. All isolates were susceptible to amikacin, vancomycin, teicoplanin, daptomycin, and linezolid.

After molecular study, the *mecA* gene was detected in all strains. Of the seven erythromycin-resistant isolates, only one harbored the *msrA* gene. This gene encodes an ATP-binding cassette (ABC) efflux pump that mediates resistance to 14- and 15-membered macrolides, such as erythromycin and clarithromycin, as well as to streptogramin B antibiotics. Notably, *msrA* does not confer resistance to clindamycin. No other macrolide resistance determinants were detected among the isolates. The *acc (6′)-Ib* acetyltransferase gene was detected in seven isolates. The *acc(6′)-Ib* gene is associated with resistance to the aminoglycosides kanamycin, tobramycin, and amikacin. However, only four of the eight isolates that expressed this gene were resistant to tobramycin, and none of them expressed resistance to amikacin. Furthermore, four isolates demonstrated resistance to gentamicin, yet no additional resistance genes were identified for this group of antibiotics. Resistance to kanamycin was not tested. No fluoroquinolone resistance genes were detected. Detailed antibiotic susceptibility and genotypic data are presented in Table 2.

MRSA nasal colonization was detected in 43.3% of patients (13/30; 95% CI: 27.4–60.8%) and 27.3% of healthcare workers (3/11; 95% CI: 6.0–61.0%). The difference between these groups was not statistically significant (Fisher’s exact test, *p* = 0.733). Eight patients with confirmed MRSA colonization were admitted to the Leprosy Ward and had a history of empirical cloxacillin treatment for ulcers in the previous three months. The remaining eight patients were from the Outpatient Department (4), Leprosy Ward (2), Maternity Ward (1), and Emergency Department (1). Data for all individuals with MRSA isolation are shown in Table 3.

### 2.3. Rectal Colonization Study

Rectal swabs were collected from 48 patients (28 adults and 20 children). MDR bacteria were isolated from 21 patients (17 pediatric and 4 adults). Data for these patients are shown in Table 4. The overall prevalence of rectal colonization was 43.7%.

*Escherichia coli* was the most frequently isolated microorganism (37.5%), followed by *Enterococcus faecium* (25%) and *Klebsiella pneumoniae* (10.4%).

Nineteen MDR *E. coli* strains were isolated from 18 patients. Twelve strains co-produced NDM carbapenemase and CTX-M type ESBL (one also with AmpC). One isolated carried OXA-48 carbapenemase, two carried CTX-M and AmpC, and four *E. coli* carried CTX-M.

All NDM-producing strains (12) were susceptible only to colistin, amikacin, and tigecycline. Molecular analysis identified the aminoglycoside resistance gene *aac(6′)-Ib* in all 12 strains. This gene has been associated with resistance to tobramycin, kanamycin, and moderate resistance to amikacin. However, resistance to gentamicin and tobramycin was detected phenotypically in only 11 strains, and no isolates were found to be resistant to amikacin. In all strains, *sul1* gene was detected. This gene is related to trimethoprim-sulfamethoxazole resistance, and all the strains were phenotypically resistant to this antibiotic. The macrolide resistance gene *ermB* was detected in all isolates; this is not common, since this gene is usually expressed in Gram-positive cocci. The presence of this gene in *E. coli* strains may be due to horizontal gene transfer. Finally, the gene for resistance to chloramphenicol *catB3* was detected in three isolates, although all isolates were resistant to this antibiotic.

ESBL-producing *E. coli* strains (four) were resistant to amoxicillin-clavulanic acid (75%), ceftazidime (100%), cefotaxime (100%), ciprofloxacin (25%), and trimethoprim-sulfamethoxazole (75%). *E. coli* ESBL and AmpC co-producers were resistant to amoxicillin-clavulanic acid, piperacillin-tazobactam, cefoxitin, ceftazidime, cefotaxime, ciprofloxacin, gentamicin, tobramycin, and trimethoprim-sulfamethoxazole.

Five ESBL-producing *Klebsiella pneumoniae* isolates were detected. The *bla*_CTX-M_ gene was detected in all isolates, and all of them were resistant to amoxicillin-clavulanic acid, ceftazidime and cefotaxime. One isolate was resistant to piperacillin-tazobactam. Regarding other antibiotic families, the five strains were susceptible to all antibiotics except three isolates resistant to ciprofloxacin and four to trimethoprim-sulfamethoxazole. No acquired resistance genes to these antibiotics were detected genotypically.

Twelve vancomycin-resistant *E. faecium* strains were isolated from 12 patients, all with the *vanA* genotype. All isolates were resistant to ampicillin, with an MIC greater than 8 mg/L. Eleven isolates were resistant to teicoplanin; one isolate was susceptible, with an MIC of 0.5 mg/L. All 12 isolates were susceptible to linezolid. Although enterococci are intrinsically resistant to macrolides, the acquired resistance gene *ermB* was detected in 11 of these isolates. The resistance genes of all strains in the rectal colonization study are listed in Table 5.

**Figure 1:**
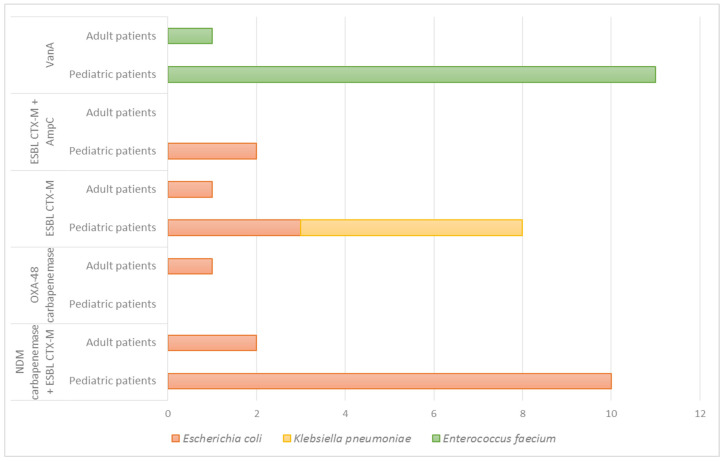
Mechanisms of acquired resistance in multidrug-resistant microorganisms isolated during the study of rectal colonization, categorized by age group and bacterial species.

Rectal colonization by multidrug-resistant (MDR) bacteria was detected in 14.3% of adult patients (4/28; 95% CI: 5.7–31.5%) and 85% of pediatric patients (17/20; 95% CI: 62.1–96.8%) (*p* = 0.0001). In adult patients, VRE (*n* = 1), *E. coli* CTX-M-type ESBL producer (*n* = 1), and *E. coli* NDM and CTX-M co-producers (*n* = 2), one of which also carried OXA-48, were identified. In pediatric patients, *E. coli* NDM and CTX-M co-producers were detected in 50% (10/20), and VRE was isolated in 55% (11/20) (Figure 1).

Thirty-five percent of the patients carried both microorganisms. In addition, *K. pneumoniae* carrying CTX-M was isolated in five patients (25%).

**Figure 2:**
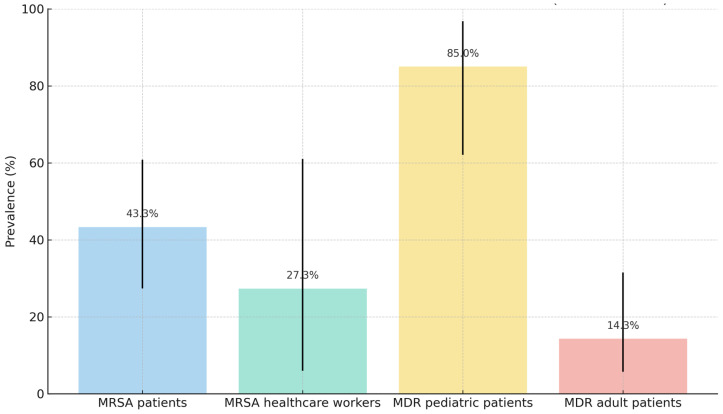
Prevalence and 95% confidence intervals of MRSA nasal colonization and MDR rectal colonization among patients and healthcare workers.

Summary statistics for antimicrobial resistance profiles of key isolates and colonization rates are presented in Table 6 and Figure 2.

## 3. Discussion

This study investigated the rates of nasal colonization by MRSA and rectal colonization by beta-lactamase-producing Enterobacterales and VRE in patients and healthcare workers at Gambo General Hospital. The overall prevalence of MRSA was 39%, with colonization rates of 43% in patients and 27% in healthcare workers. These rates exceed those reported in other studies [17,18,19,20]. Two meta-analyses [17,18] conducted in the last decade showed that the pooled prevalence of MRSA colonization in Ethiopia was 32.5% and 10.94%, respectively, while in the Oromia region, the percentages were 39.1% and 21.28%. A total prevalence of 31.6% was found in the capital, Addis Ababa [17]. The prevalence of colonization varies considerably across different regions of the world. Rates range from 9.1% in Europe and 10% in Oceania, to higher percentages of 22% in North America and 25.6% in Asia [21]. The most recent studies in Europe report a prevalence of 28.51% in Spain [22], 5.5% in Germany [23], 2–5% in Switzerland [24], and 2% in the Netherlands [25]. These rates are much lower than those reported in Ethiopia and other African countries, excluding Kenya, where the prevalence was reported to be less than 3% [26].

The high MRSA prevalence in this study may be influenced by multiple risk factors exacerbated by poverty in LMICs, such as HIV infection, residence in rural areas, and admission to surgical facilities [17,27]. The excessive and uncontrolled use of antibiotics in these settings contributes to the high global prevalence of AMR [17,28]. All patients with MRSA in this study were receiving or had recently received cloxacillin for ulcers, which may suggest a potential association between antibiotic exposure and MRSA colonization [29,30,31,32].

A significant proportion of healthcare workers (27%) were MRSA carriers, which could represent a potential risk for transmission to patients and other staff. Measures such as identifying colonized individuals, promoting hand hygiene, and implementing other precautions are crucial for reducing MRSA transmission [33]. Although the sample size was small, the percentage of healthcare workers colonized by MRSA in our study is higher than in other recent studies conducted in Ethiopia, where the prevalence ranged between 4.8% [34] and 11.2% [35].

The rectal colonization study revealed a high prevalence of MDR bacteria (43.7%), particularly in pediatric patients. A concerning finding was the isolation of *E. coli* strains co-producing NDM carbapenemase and CTX-M ESBL from 25% of patients. Fecal colonization by carbapenemase-producing Enterobacterales has been reported with increasing frequency in Ethiopia, ranging from 2.01% [36] to 7.3% [37]. To our knowledge, this study is one of the first to detect NDM carbapenemase and CTX-M ESBL co-producing *E. coli* strains in rectal colonization samples in a hospital in Ethiopia. The isolation of NDM-producing *E. coli* strains has increased in recent years also in European countries [26]. However, the global prevalence of carbapenemase-producing Enterobacterales remains relatively low, staying below 7% in Asian countries, and even lower in Europe and North America, with prevalences of 0.2% and 5%, respectively [21]. In other African countries, such as Kenya, the prevalence of carbapenemase-producing Enterobacteriaceae is reported to be 7% in rural hospitals [26].

The high prevalence of carbapenemase-producing strains in pediatric patients (83.3%; 10/12) may be attributed to factors such as overcrowded conditions and poor hand hygiene practices. The uniform susceptibility profile of the NDM-producing strains suggests a possible clonal spread [38].

A total of 24 Enterobacterales strains were isolated, 100% of which were ESBL producers. Of these 24 isolates, 79.2% were *E. coli*, and 20.8% were identified as *K. pneumoniae*. Other studies conducted in Ethiopian hospitals calculated a percentage of ESBL-producing Enterobacterales in colonization samples ranging from 17 to 52% relative to the total number of isolates [36,37,39]. Thus, the estimated proportion of ESBL-producing *Enterobacteriaceae* in East African hospitals is 42%, which is much higher than the estimated proportion in European countries [39].

VRE was isolated in 25% of patients. This percentage is much higher than in other studies where VRE was detected in 1.33% of patients [40]. Studies conducted in other African countries showed VRE colonization rates were over 20% particularly in Egypt [41], Sudan [42], Algeria [43] and South Africa [44]. In Western countries, the prevalence of VRE is 4% in the United States, compared to below 1% in European countries [21]. All our isolates expressed the *vanA* genotype, which is characterized by high levels of resistance to vancomycin and teicoplanin. Of the 12 isolates, 11 were indeed resistant to vancomycin (MIC > 256 mg/L) and teicoplanin (MIC > 12 mg/L), but one isolate showed resistance to vancomycin with a MIC > 256 mg/L and susceptibility to teicoplanin with a MIC of 0.5 mg/L. This phenomenon is called ‘*vanA* genotype–*vanB* phenotype’ and has been described in other African countries [30].

The high prevalence of MDR bacteria in the rectum of patients, many of whom had not received antibiotic treatment, suggest a high dissemination capacity exacerbated by poor hygiene practices and overcrowded conditions.

This study has several limitations that must be acknowledged. First, the relatively small sample size (*n* = 89), particularly when stratified by colonization site and bacterial species, limits the statistical power of the findings and increases the risk of both type I and type II errors. As a result, the associations observed should be interpreted with caution. Second, conducting the study in a single rural center may have introduced sampling bias and restricted the extrapolation of results to other settings, particularly urban or tertiary care centers. Third, the rectal colonization study used different sample types (rectal swabs, vagino-rectal swabs, and stools). Although previous studies have found no difference in bacterial recovery rates between rectal swabs and stool samples, this variation may have influenced detection sensitivity.

Also, it is important to note that eight MRSA isolates could not be transported to our hospital laboratory, so they could not be included in the identification study, antibiotic susceptibility testing or genotypic analysis. Finally, due to the limited sample size, it was not possible to conduct a multivariable analysis controlling for potential confounders such as age, underlying disease, and antimicrobial exposure. Future studies with larger, multicenter cohorts and standardized sampling protocols are needed to validate these findings and strengthen their generalizability.

## 4. Materials and Methods

Nasal exudates were collected using sterile swabs inserted in both nostrils. Rectal colonization was assessed by collecting three types of specimens, depending on the study population. Rectal swabs were collected from adults using sterile swabs with Stuart transport medium (CliniswabTS^®^, VWR International, and EUROTUBO^®^ Tube with Transport Media, Deltalab, Barcelona, Spain). The swab was gently inserted 2–3 cm into the rectum and rotated to ensure adequate contact with the mucosal surface. In pregnant women, combined vaginal-rectal samples were obtained, where a sterile swab (CliniswabTS^®^, VWR International, Barcelona, Spain) was first inserted into the lower vaginal area (without speculum use), and then gently introduced into the rectum through the anal sphincter. The swab was rotated in both sites to ensure adequate sample collection before being placed into the transport medium for laboratory processing. In pediatric patients and young children for whom rectal swabbing was not feasible, fresh stool samples were collected directly, and the samples were placed in sterile containers and transferred to the laboratory within 4–6 h of collection.

The sample collection period was from 26 May to 9 June 2024.

Patient demographic data were obtained from medical records and interviews. Verbal informed consent was obtained from all subjects. No formal sample size calculation or power analysis was conducted, as this was an exploratory study carried out during a limited field stay. The sample size was determined by the number of eligible participants who consented to be included during the study period. The inclusion criterion was all patients seen in consultation or admitted to the hospital who consented to participate and from whom a sample could be collected. The exclusion criterion was unwillingness to participate in the study.

Nasal swabs were placed on CHROMID^®^ MRSA agar (bioMérieux, Marcy-l’Étoile, France) for MRSA colony detection. Rectal colonization samples were plated on CHROMID^®^ VRE Agar (bioMérieux, Marcy-l’Étoile, France) for the detection of VRE and CHROMID^®^ CARB/OXA Agar (bioMérieux, Marcy-l’Étoile, France) for the detection of CPE. Samples were processed within 24 h of collection and incubated at 37 °C for 24 h. A negative control was performed on CHROMID^®^ CARB/OXA agar plates with an *Escherichia coli* strain without resistance mechanisms.

After initial isolation on selective chromogenic media, bacterial colonies were preserved using Cryoinstant Color Mix cryotubes (Deltalab SL, Barcelona, Spain), then frozen and later transported under cold chain conditions to Madrid (Spain) for identification, antimicrobial susceptibility testing, and genotypic analysis.

Isolates were identified by MALDI-TOF mass spectrometry (Bruker Daltonics GmbH, Leipzig, Germany), and antibiotic susceptibility testing was performed using the automated MicroScan Walkaway system (Beckman Coulter, Madrid, Spain). Interpretation of results followed the European Committee for Antimicrobial Susceptibility Testing (EUCAST) guidelines [45]. Isolates identified as *Escherichia coli* were confirmed through biochemical profiling (lactose fermentation, indole production) to exclude potential misidentification as *Shigella* species, which share >99% 16S rRNA sequence homology but lack characteristic *E. coli* metabolic capabilities.

The initial study of beta-lactamase production in Enterobacteriaceae was conducted using the immunochromographic tests NG-Test^®^/CTX-M Multi (NG-Biotech Laboratories, Guipry, France) for CTX-M type (BLEE) and NG-Test^®^/CARBA-5 (NG-Biotech Laboratories, Guipry, France) for carbapenemase production, including KPC, OXA-48, VIM, IMP, and NDM. VRE isolates were tested for MIC against vancomycin and teicoplanin using concentration gradient strips (Liofilchem, Roseto degli Abruzzi, Italy). Once the presumptive presence of an antibiotic resistance mechanism was confirmed, a molecular study of resistance genes was performed in all MDR bacterial strains using the Multidrug Resistance (MDR) Direct Flow Chip Kit (Vitro, Master Diagnóstica, Granada, Spain) directly from bacterial colonies. The MDR Direct Flow Chip (Vitro S.A., Spain) is a molecular diagnostic kit based on multiplex PCR amplification followed by reverse hybridization on a DNA microarray membrane. This assay enables the simultaneous detection of five bacterial species (*S. aureus*, *K. pneumoniae*, *Pseudomonas aeruginosa*, *E. coli* and *Acinetobacter baumannii*) and a wide range of antimicrobial resistance genes directly from bacterial isolates, including 55 AMR determinants for beta-lactams (23), quinolones (13), aminoglycosides (5), macrolides (5), sulfonamides (3), colistin (2), vancomycin (2), chloramphenicol (1), and linezolid (1). The biotin-labeled PCR products hybridize with specific oligonucleotide probes immobilized on the chip membrane, and hybridization is detected via a colorimetric reaction [46,47].

### Data Analysis

Statistical analysis was performed using SPSS^®^ v27.0 (IBM Corp., Armonk, NY, USA). Continuous variables were presented as medians and interquartile ranges (IQRs), and categorical variables were presented as proportions. Proportions are presented with 95% confidence intervals calculated using the Wilson method, and group comparisons were performed using χ^2^ test or Fisher’s exact test. A *p*-value ≤ 0.05 was considered statistically significant for these comparisons.

## 5. Conclusions

This study provides crucial evidence of a substantial burden of multidrug-resistant organisms, including MRSA, NDM/CTX-M co-producing Enterobacterales, and VRE, within Gambo General Hospital, a rural Ethiopian hospital setting. These findings underscore the critical need for infection prevention strategies and strengthened antimicrobial stewardship efforts. Specific interventions, such as improved hand hygiene, continuous surveillance of multidrug-resistant bacteria, and prudent antibiotic use, are crucial for mitigating the spread of antimicrobial resistance in high-risk settings. Looking ahead, it is imperative to adopt collaborative, locally tailored approaches to enable effective monitoring and control of resistance in resource-limited hospitals. This requires equipping these centers with adequate resources and promoting teamwork, as well as the ongoing training and education of healthcare professionals. These are key elements for implementing sustainable, context-specific strategies to combat antimicrobial resistance effectively.

## Figures and Tables

**Table 1:** Demographic characteristics of study participants.

Study	Group	*n*	Median Age (IQR)	Sex
Nasal Colonization	Patients	30	25.5 (19.25–40)	10 males; 20 females
	Healthcare workers	11	28 (25–35)	7 males; 4 females
Rectal Colonization	Pediatric patients	20	1.2 (0.75–4)	20 males; 8 females
	Adult patients	28	25 (14–40)	1 males; 27 females

**Table 2:** Antimicrobial susceptibility data and genotypic data of methicillin-resistant *Staphylococcus aureus* isolates.

	Antimicrobial Susceptibility Testing (MIC g/dL) *													Genotypic Analysis		
Order	OX	CFXS	VNC	TEC	LIN	DAP	ERY	CMN	CIP	LVX	GMN	TMN	AKN	*mecA*	*acc(6* *′)-lb*	*msrA*
1	- **	*-*	*-*	*-*	*-*	*-*	*-*	*-*	*-*	*-*	*-*	*-*	*-*	-	-	-
2	*-*	*-*	*-*	*-*	*-*	*-*	*-*	*-*	*-*	*-*	*-*	*-*	*-*	-	-	-
3	*-*	*-*	*-*	*-*	*-*	*-*	*-*	*-*	*-*	*-*	*-*	*-*	*-*	-	-	-
4	*-*	*-*	*-*	*-*	*-*	*-*	*-*	*-*	*-*	*-*	*-*	*-*	*-*	-	-	-
5	*-*	*-*	*-*	*-*	*-*	*-*	*-*	*-*	*-*	*-*	*-*	*-*	*-*	-	-	-
6	*-*	*-*	*-*	*-*	*-*	*-*	*-*	*-*	*-*	*-*	*-*	*-*	*-*	-	-	-
7	*-*	*-*	*-*	*-*	*-*	*-*	*-*	*-*	*-*	*-*	*-*	*-*	*-*	-	-	-
8	*-*	*-*	*-*	*-*	*-*	*-*	*-*	*-*	*-*	*-*				-	-	-
9	>2	>4	1	≤1	2	≤1	>4	≤0.25	2	2	4	8	≤8	+ ***	+	−
10	>2	>4	1	≤1	2	≤1	>4	≤0.25	>2	4	>8	>8	≤8	+	+	−
11	>2	>4	1	≤1	≤1	≤1	>4	≤0.25	≤1	≤1	≤1	≤1	≤8	+	+	−
12	>2	>4	1	1	2	≤1	>4	≤0.25	>2	4	>8	8	≤8	+	+	−
13	>2	>4	1	≤1	≤1	≤1	>4	≤0.25	>2	4	>8	>8	≤8	+	+	−
14	>2	>4	1	≤1	≤1	≤1	>4	≤0.25	≤1	≤1	≤1	≤1	≤8	+	+	−
15	>2	>4	1	≤1	≤1	≤1	>4	≤0.25	>2	>4	≤1	≤1	≤8	+	+	+
16	>2	>4	1	≤1	2	≤1	>4	≤0.25	≤1	≤1	≤1	≤1	≤8	+	−	−

* OX: Oxacillin; CFXS: Cefoxitin; VNC: Vancomycin; TEC: Teicoplanin; LIN: Linezolid; DAP: Daptomycin; ERY: Erythromycin; CMN: Clindamycin; CIP: Ciprofloxacin; LVX: Levofloxacin; GMN: Gentamicin; TMN: Tobramycin; AKN: Amikacin. ** -: No data available. *** +: Positive result; −: Negative result.

**Table 3:** Demographic data of patients with methicillin-resistant *Staphylococcus aureus* isolation.

Order	Patient/ Healthcare Workers	Age (Years)	Gender	Residential Area	Hospital Department (Admission/Work)	History of Empirical Cloxacillin Treatment for Ulcers in the Previous 3 Months	Genotypic Analysis	Antimicrobial Susceptibility Testing
1	Healthcare worker	30	M	Kore	Maternity Ward	No	No	No
2	Patient	35	F	Negele	OPD *	Yes	No	No
3	Patient	48	M	Kore	Leprosy Ward	Yes	No	No
4	Patient	28	F	Kore	Leprosy Ward	Yes	No	No
5	Healthcare worker	25	M	Kore	OPD	No	No	No
6	Healthcare worker	60	M	Kore	OPD	No	No	No
7	Patient	40	F	Kore	OPD	Yes	No	No
8	Patient	51	F	Lepis	Emergency Department	No	No	No
9	Patient	72	M	Kokosa	Leprosy Ward	Yes	Yes	Yes
10	Patient	24	F	Kabira	Leprosy Ward	Yes	Yes	Yes
11	Patient	46	F	Kore	Leprosy Ward	Yes	Yes	Yes
12	Patient	81	M	Rigelu	Leprosy Ward	Yes	Yes	Yes
13	Patient	63	F	Hogiso	Leprosy Ward	Yes	Yes	Yes
14	Patient	55	M	Kore	Leprosy Ward	Yes	Yes	Yes
15	Patient	66	M	Hogiso	Leprosy Ward	Yes	Yes	Yes
16	Patient	51	M	Kore	Leprosy Ward	Yes	Yes	Yes

* OPD: Outpatient Department.

**Table 4:** Data for patients with positive results in the rectal colonization study.

Patient	Age	Gender	Residential Area	Admission Department	History of Empirical Antibiotic in the Previous 3 Months	Sample	Isolate
1	1 months	M	Basaku Ilala	Pediatric Ward	Ceftriaxone, Cloxacillin	Stool	*E. faecium*
2	3 years old	M	Ashoka	Pediatric Ward	Ceftriaxone, Cloxacillin	Stool	*E. coli*
3	11 months	M	Kore	Pediatric Ward	Ceftriaxone, Ampicillin, Gentamicin	Stool	*E. coli* *K. pneumoniae* *E. faecium*
4	14 months	F	Kore	Pediatric Ward	Ceftriaxone	Stool	*E. coli* *E. faecium*
5	5 months	M	Lepis	Pediatric Ward	Ampicillin, Gentamicin, Ceftriaxone, Cloxacillin	Stool	*E. coli* *E. faecium*
6	6 months	M	Kore	Pediatric Ward	Ceftriaxone, Cloxacillin, Gentamicin	Stool	*E. coli* *E. faecium*
7	2 years old 7 months	M	Basaku Ilala	Pediatric Ward	Ceftriaxone, Gentamicin	Stool	*E. coli* *K. pneumoniae*
8	13 years old	M	Lepis	Pediatric Ward	Ceftriaxone	Stool	*E. coli*
9	35 years old	F	Negele	OPD *	Cloxacillin	Rectal exudate	*E. coli*
10	28 years old	F	Kore	Leprosy Ward	Cloxacillin	Rectal exudate	*E. coli* *E. coli*
11	4 years old	M	Basaku Ilala	Pediatric Ward	Ceftriaxone, Gentamicin, Ampicillin	Stool	*E. coli* *K. pneumoniae* *E. faecium*
12	4 years old	M	Basaku Ilala	Pediatric Ward	Ampicillin, Gentamicin	Stool	*K. pneumoniae*
13	9 months	F	Lepis	Pediatric Ward	Ceftriaxone, Vancomycin	Stool	*E. coli*
14	11 months	M	Aga Nia	Pediatric Ward	Ceftriaxone, Gentamicin, Ampicillin	Stool	*E. coli* *K. pneumoniae* *E. faecium*
15	18 months	F	Kore	Pediatric Ward	Ceftriaxone	Stool	*E. coli* *E. faecium*
16	11 years old	F	Kore	Pediatric Ward	Ceftriaxone	Stool	*E. coli*
17	8 months	F	Kore	Pediatric Ward	Ceftriaxone	Stool	*E. coli* *E. faecium*
18	3 years old	F	Kore	Pediatric Ward	Ceftriaxone	Stool	*E. coli* *E. faecium*
19	11 months	M	Lepis	Pediatric Ward	Ceftriaxone, Amoxicillin	Stool	*E. coli* *E. faecium*
20	40 years old	F	Kore	OPD	Cloxacillin, Amoxicillin, Ceftriaxone, Ciprofloxacin	Rectal exudate	*E. coli*
21	18 years old	F	Aga Nia	Antenatal Department	None	Vagino-rectal exudate	*E. faecium*

* OPD: Outpatient Department.

**Table 5:** Genotypic study of the strains isolated in the rectal colonization study ordered by patient.

Patient	Isolate	*bla*_CTX-M_	*bla*_CMY_	*bla*_NDM_	*bla*_oxa48 like_	*sul1*	*sul2*	*qnrS*	*ermB*	*aac (6′)-Ib*	*catB3*	*vanA*
1	*E. faecium*	−	−	−	−	−	−	−	+	−	−	+
2	*E. coli*	+	−	+	−	+	−	−	+	+	−	−
3	*E. coli*	+	−	−	−	−	−	−	−	−	−	−
	*K. pneumoniae*	+	−	−	−	−	−	−	−	−	−	−
	*E. faecium*	−	−	−	−	−	−	−	−	−	−	+
4	*E. coli*	+	+	+	−	+	−	−	+	−	−	−
	*E. faecium*	−	−	−	−	−	−	−	+	−	−	+
5	*E. coli*	+	+	−	−	−	−	−	+	+	−	−
	*E. faecium*	−	−	−	−	−	−	−	+	−	−	+
6	*E. coli*	+	−	+	−	+	−	−	+	+	−	−
	*E. faecium*	−	−	−	−	−	−	−	+	−	−	+
7	*E. coli*	+	−	−	−	−	−	−	−	−	−	−
	*K. pneumoniae*	+	−	−	−	−	−	−	−	−	−	−
8	*E. coli*	+	−	+	−	+	−	−	+	+	−	−
9	*E. coli*	+	−	+	−	+	−	−	+	+	−	−
10	*E. coli*	+	−	+	−	+	−	−	+	+	−	−
	*E. coli*	−	−	−	+	−	−	−	−	−	−	−
11	*E. coli*	+	−	+	−	+	−	−	+	+	−	−
	*K. pneumoniae*	+	−	−	−	−	−	−	−	−	−	−
	*E. faecium*	−	−	−	−	−	−	−	+	−	−	+
12	*K. pneumoniae*	+	−	−	−	−	−	−	−	−	−	−
13	*E. coli*	+	−	+	−	+	−	−	+	+	−	−
14	*E. coli*	+	−	+	−	+	−	−	+	+	+	−
	*K. pneumoniae*	+	−	−	−	−	−	−	−	−	−	−
	*E. faecium*	−	−	−	−	−	−	−	+	−	−	+
15	*E. coli*	+	−	+	−	+	−	−	+	+	+	−
	*E. faecium*	−	−	−	−	−	−	−	+	−	−	+
16	*E. coli*	+	−	−	−	−	−	−	−	−	−	−
17	*E. coli*	+	−	+	−	+	−	−	+	+	+	−
	*E. faecium*	−	−	−	−	−	−	−	+	−	−	+
18	*E. coli*	+	+	−	−	−	−	−	+	+	−	−
	*E. faecium*	−	−	−	−	−	−	−	+	−	−	+
19	*E. coli*	+	−	+	−	+	−	−	+	+	−	−
	*E. faecium*	−	−	−	−	−	−	−	+	−	−	+
20	*E. coli*	+	−	−	−	−	−	−	−	−	−	−
21	*E. faecium*	−	−	−	−	−	−	−	+	−	−	+

+: Positive result; −: Negative result.

**Table 6:** Antimicrobial resistance profiles of key isolates.

Organism	*n*	Main Resistance Genes Detected	Most Frequent Phenotypic Resistances
MRSA (*S. aureus*)	8	*mecA*, *msrA*, *acc(6′)-Ib*	Oxacillin, cefoxitin, erythromycin, clindamycin
MDR *E. coli*	19	*bla*_NDM_, *bla*_CTX-M_, *bla*_CMY_, *bla*_OXA-48_, *ermB*	Third-gen cephalosporins, carbapenems, TMP-SMX
MDR *K. pneumoniae*	5	*bla*_CTX-M_	Third-gen cephalosporins, gentamicin, tobramycin
VRE (*E. faecium*)	12	*vanA*, *ermB*	Vancomycin, teicoplanin

## Data Availability

The original contributions presented in this study are included in the article. Further inquiries can be directed to the corresponding authors.

## Abbreviations

The following abbreviations are used in this manuscript:

MDR	Multidrug-resistant
MRSA	Methicillin-resistant *Staphylococcus aureus*
VRE	Vancomycin-resistant *Enterococcus* spp.
CPE	Carbapenemase-producing *Enterobacteriaceae*
AMR	Antimicrobial resistance
WHO	World Health Organization
ESBL	Extended-spectrum beta-lactamase
LMICs	Low- and middle-income countries
MIC	Minimum inhibitory concentration
IQRs	Interquartile ranges
